# Characterization of oral and cloacal microbial communities of wild and rehabilitated loggerhead sea turtles (*Caretta caretta*)

**DOI:** 10.1186/s42523-021-00120-5

**Published:** 2021-09-03

**Authors:** Klara Filek, Adriana Trotta, Romana Gračan, Antonio Di Bello, Marialaura Corrente, Sunčica Bosak

**Affiliations:** 1 Department of Biology, Faculty of Science, University of Zagreb, Rooseveltov trg 6, 10 000 Zagreb, Croatia; 2 Department of Veterinary Medicine, University of Bari “Aldo Moro”, Str. Prov. Per Casamassima Km 3, 70010 Valenzano, BA Italy

**Keywords:** Microbiota, Bacterial diversity, Reptile, Rehabilitation, Adriatic Sea, Conservation

## Abstract

**Background:**

Microbial communities of wild animals are being increasingly investigated to provide information about the hosts’ biology and promote conservation. Loggerhead sea turtles (*Caretta caretta*) are a keystone species in marine ecosystems and are considered vulnerable in the IUCN Red List, which led to growing efforts in sea turtle conservation by rescue centers around the world. Understanding the microbial communities of sea turtles in the wild and how affected they are by captivity, is one of the stepping stones in improving the conservation efforts. Describing oral and cloacal microbiota of wild animals could shed light on the previously unknown aspects of sea turtle holobiont biology, ecology, and contribute to best practices for husbandry conditions.

**Results:**

We describe the oral and cloacal microbiota of Mediterranean loggerhead sea turtles by 16S rRNA gene sequencing to compare the microbial communities of wild *versus* turtles in, or after, rehabilitation at the Adriatic Sea rescue centers and clinics. Our results show that the oral microbiota is more sensitive to environmental shifts than the cloacal microbiota, and that it does retain a portion of microbial taxa regardless of the shift from the wild and into rehabilitation. Additionally, Proteobacteria and Bacteroidetes dominated oral and cloacal microbiota, while Kiritimatiellaeota were abundant in cloacal samples. Unclassified reads were abundant in the aforementioned groups, which indicates high incidence of yet undiscovered bacteria of the marine reptile microbial communities.

**Conclusions:**

We provide the first insights into the oral microbial communities of wild and rehabilitated loggerhead sea turtles, and establish a framework for quick and non-invasive sampling of oral and cloacal microbial communities, useful for the expansion of the sample collection in wild loggerhead sea turtles. Finally, our investigation of effects of captivity on the gut-associated microbial community provides a baseline for studying the impact of husbandry conditions on turtles’ health and survival upon their return to the wild.

**Supplementary Information:**

The online version contains supplementary material available at 10.1186/s42523-021-00120-5.

## Background

Microbial communities associated with vertebrates can influence host’s evolution, development, immune system maturation, physiology, nutrient acquisition, health and disease [1, 2]. It is estimated that the host’s collection of bacteria could contain at least 100 times the genes as in the host’s genome, often adding to the metabolic functions’ repertoire, e.g. biochemical pathways in nutrient acquisition [3]. Moreover, we can consider the host and its microbial commensals as a distinct biological entity (holobiont and hologenome) susceptible to the processes of natural selection [4, 5].

Most studies of microbial communities have focused on the distal gut of humans or captive mammals [2, 6] but there are recent growing efforts in investigations of free-ranging wild animals. Wild animals are sensitive to environmental perturbations caused by climate change and anthropogenic habitat disruption, therefore investigating wild animal-associated microbial communities contributes to improving existing conservation efforts [7, 8]. Current research covering major vertebrate groups reveal evidence for co-phylogeny of mammals and their microbial communities, microbiome convergence in birds and bats, while microbial assemblages of non-mammalian hosts (e.g. reptiles) are mostly influenced through diet and the environment [9, 10]. Marine animals are permanently immersed in seawater environment, making the microbial dynamics different from those of land-dwelling animals [11]. As expected, marine mammals have been the focus of most vertebrate microbial community studies that undertook a wider sampling effort of body sites other than the distal gut or feces [12–16]. In comparison to other vertebrates, reptiles are still underrepresented in studies of their bacterial communities [6, 17], especially large marine reptiles, such as sea turtles. Sea turtles are large-bodied, long-lived marine top predators, considered as a keystone species, with critical roles in ecosystem processes such as bioturbation, bioaccumulation, energy flow, trophic status and mineral cycling [18]. Loss of foraging and nesting sites, increasing global temperatures, and bycatch are major threats for sea turtles’ survival. Currently, there are seven extant sea turtle species listed on the IUCN Red List of Threatened Species [19]: Kemp’s ridley (*Lepidochelys kempii*) and hawksbill sea turtles (*Eretmochelys imbricata*) are critically endangered; the green turtle (*Chelonia mydas*) is considered to be endangered; loggerhead (*Caretta caretta*), olive ridley (*Lepidochelys olivacea*) and leatherback (*Dermochelys coriacea*) sea turtles are listed as vulnerable, while data are deficient for the flatback sea turtle (*Natator depressus*). The efforts of sea turtle rescue and rehabilitation initiatives facilitate access for sea turtle-focused research [20] and, consequently, studies on microbial communities of sea turtles are increasing.

To date, microbial assemblages of the sea turtle gut have been described by sequencing the 16S rRNA genes of fecal or cloacal samples in wild, stranded [21, 22], and rehabilitated green sea turtles [23, 24], in juveniles undergoing an ontogenetic shift from pelagic to neritic habitats [25, 26], and mucosa-associated bacterial communities in stranded green turtles [27]. Additionally, there are reports on the gut microbiota of Kemp’s ridley turtles undergoing rehabilitation [28] and nesting flatback turtles [29, 30]. Loggerhead sea turtles’ fecal and gut microbial communities have been studied mostly in stranded animals or undergoing rehabilitation in the Mediterranean Sea [31–33] with recent reports on nesting females of the USA and Australian populations [30, 34]. Furthermore, Scheelings and colleagues have performed one of the most comprehensive studies on the distal gut microbial communities of all seven species of the sea turtles reporting phylogenetic aspects of sea turtle microbiome evolution [34].

The focus of this study is on the loggerhead sea turtles’ oral and distal gut microbiota in both recently caught and turtles undergoing rehabilitation at the Adriatic Sea turtle rescue centers. In addition to distal gut (cloacal) samples, we sampled the buccal (oral) cavity as there are no known reports on 16S rRNA profiling for oral microbial communities in sea turtles to the date of this study. Cultivation-based approaches have shown that oral bacterial communities of loggerhead sea turtles in the Mediterranean harbor antibiotic-resistant bacterial strains and common opportunistic pathogens [35, 36]. NGS amplicon sequencing of resistant bacterial isolates showed that injured Adriatic Sea loggerheads’ wounds contain bacteria with multiple antibiotic resistance genes [37]. Aforementioned reports emphasize the idea of sea turtles as sentinel species that can be studied as indicators of marine health and pollution [35]. To fill in the gap in understanding the loggerhead sea turtle microbiota, we provide data on loggerhead oral microbial communities as the oral cavity is the first line in transitioning from external to internal environments of the turtle. The aims of this study were to describe oral and cloacal microbial communities of loggerhead sea turtles and compare them between incidentally caught or stranded and captive animals undergoing rehabilitation. Additionally, we investigated the impact of short-term rehabilitation period on loggerhead microbiota, which could clarify the dynamics of the loggerhead sea turtles’ commensal microbes in relation to the turtles’ changing environment.

## Methods

### Target population

We sampled loggerhead sea turtles from the Adriatic Sea that were found floating, stranded on beaches or incidentally caught by fishing boats and then transported to the regional veterinary clinic or rescue center: The Sea Turtle Clinic (STC) of the Department of Veterinary Medicine of University of Bari “Aldo Moro” (Italy) and the Marine Turtle Rescue Center Aquarium Pula (Croatia). Samples collected immediately upon arrival to the treatment facility are considered “wild” as they were taken close to the time of turtle capture and marked as “before” samples in further analyses and text. All turtles were examined for injuries and relevant information were collected during sampling. Healthy individuals were released within 24 h, while others were kept under observation (“short-term rehabilitation”) or longer rehabilitation until recovered from injuries. List of sampled turtles is presented in Table 1 with an indication of release day.

**Table 1:** Information on sampled loggerhead sea turtles and their condition at the time of admission to the rescue centers in the Adriatic Sea

Turtle ID	Origin	Sampling date (YYYY-MM-DD)	Days before sampling	Sampling site			CCL (cm)	Weight (kg)	Sex	Life stage	Reason for admission to rescue center
				Oral	Cloacal	Water					
ID010	CRO, Korčula	2018-12-11	3	–	+	–	NA	40	F	adult	Old head injury; weight was measured prior to release 2019-11-04
		2019-04-08	121	–	+	+	69.7	46.5			
ID019	IT, Barletta	2019-01-09	0	+	+	–	50.7	31	ND	juvenile	Trawling (32 m depth), treated for gas embolism
		2019-01-11	2^R^	+	+	–					
ID022	IT, Barletta-Trani	2019-01-10	0^R^	+	+	–	72	42	M	adult	Trawling (22 m depth), released during the day
ID023	IT, Trani	2019-01-11	13	+	+	–	64	30.6	ND	juvenile	Trawling (36 m depth) hospitalized at the WWF center in fresh water (7 days leeches removal treatment)
ID026	IT, Molfetta	2019-01-21	6^R^	+	+	+	64.5	32.1	M	adult	Found at the beach, not good clinical status
ID028	IT, Barletta	2019-01-17	0	+	+	–	74.5	46	F	adult	Trawling (34 m depth), gas embolism
ID030	IT, Barletta	2019-01-17	0	+	+	–	63	29.5	ND	juvenile	Trawling (34 m depth), gas embolism
		2019-01-21	4^R^	+	+	+					
ID034	IT, Bisceglie	2019-01-22	0^R^	–	+	–	72	45.6	F	adult	Trawling (40 m depth), good clinical status
ID040	IT, Barletta-Trani	2019-01-28	4^R^	–	+	–	66	35.2	ND	juvenile	Trawling (40 m depth), good clinical status
ID041	IT, Barletta-Trani	2019-01-28	4^R^	+	+	–	55.5	20	ND	juvenile	Trawling (22 m depth), gas embolism
ID042	IT, Barletta	2019-01-29	0^R^	+	+	–	67.5	36.4	ND	juvenile	Trawling (40 m depth), good clinical status
ID044	IT, Bisceglie	2019-01-29	0^R^	+	+	–	68	36.3	ND	juvenile	Trawling (30 m depth), good clinical status

Letter “R” in superscript next to the number of day before sampling indicates release into the wild on that day, if not present it means that the turtle was kept in the clinic or rescue center after the sampling

*CCL* curved carapace length, *ND* not determined

Sampling of 12 loggerhead turtles (Table 1) was performed by trained personnel during December 2018 and January 2019 in accordance with the 1975 Declaration of Helsinki, as revised in 2013, and the applicable national laws.

### Loggerheads’ enclosure description

At the STC (Italy) the hospitalized turtles were kept in individual plastic tanks (approximately 1.5 m in diameter and 1 m in depth) with clean artificial saltwater (tap water with added NaCl at least up to 35 ppt salinity). The saltwater was changed every 2–3 days, with routine tank cleaning and disinfecting between saltwater changes. At the Marine Turtle Rescue Center Aquarium Pula (Croatia), the hospitalized turtle was kept in an individual plastic tank (2 m in diameter, 1.5 m in depth) with local seawater pumped and purified through the Aquarium’s filtration systems. The tank was occasionally cleaned by scrubbing the algal overgrowth and grime off the tank walls. All turtles in the study were fed with diverse foods ranging from frozen (herring, codfish, mullet) or fresh fish food (squid, pilchard, and mackerel).

### Sample collection

The loggerheads’ cloacal and oral swab samples were collected either upon arrival of the turtle to the center (further regarded to as cloacal before, CB; oral before, OB) or within the rehabilitation period (after the turtle has spent time in the rescue center, further regarded to as cloacal rehabilitated, CR; oral rehabilitated, OR). When possible, we collected tank water during the rehabilitation period (further regarded to as tank water, W).

Oral swab samples were collected by gentle rotating of sterile dry cotton or synthetic swabs (Aptaca Nuova, Italy) on the tongue and palate mucosa, while cloacal samples were collected by inserting the swabs approximately 10 cm into the cloaca and rotating (Additional file 2: Figure S1). The swabs were collected in triplicate and stored individually in 97% ethanol at − 20 °C until DNA extraction. Samples of the tank water were collected prior to routine tank cleaning or during oral and cloacal sampling, in sterile containers and kept cool until arrival to the lab and filtering. Sampled tank water (250 ml) was vacuum filtered on a 45 mm in diameter, 0.2 μm pore-size sterile Whatman polycarbonate membrane filter (Sigma-Aldrich). Filters were carefully folded with sterile forceps and stored in 96% ethanol at − 20 °C until further processing. In total, 12 loggerhead turtles were sampled: three turtles were sampled twice (upon arrival and during rehabilitation), nine turtles were sampled once (five upon arrival, four during rehabilitation), and tank water samples were collected from three turtle enclosures (Table 1). Cloacal samples were collected from all turtles and sampling periods, while we could not obtain oral samples from three turtles (Table 1; ID010, ID034, and ID040).

### DNA extraction and sequencing

Prior to DNA extraction the ethanol was removed from the tubes by pipetting (after centrifugation) and evaporation under laminar flow hood for 24 h. DNA from the filters and swabs was extracted with the DNeasy PowerSoil Kit (Qiagen) according to the manufacturer's instructions with several modifications: (1) after transferring the swabs and filters to the PowerBead Tube the samples were incubated for 15 min at 65 °C, (2) instead of beadbeating PowerBead Tubes were vortexed horizontally for 10 min at maximum speed, and (3) all downstream incubation times at 2–8 °C were increased to 15 min. DNA was extracted from each swab and filter individually, and DNA concentrations were measured by NanoDrop ND-1000 V3.8 spectrophotometer (ThermoFisher). For samples with low DNA yield, triplicate DNA isolates were pooled together and concentrated according to the troubleshoot section of the DNeasy Powersoil Kit instructions. Extracted DNA was sent for PCR, library preparation, and 250 × 2 paired-end Illumina MiSeq v2 setup sequencing of the V3-V4 region of 16S rDNA with primers 341F_ill (5′-CCTACGGGNGGCWGCAG-3′) and 805R_ill (5′-GACTACHVGGGTATCTAATCC-3′) [38] to Microsynth (Switzerland).

### Sequence analysis

Demultiplexed sequences with removed adapters and linker sequences were obtained from the Microsynth sequencing facility and quality checked with FastQC [39]. Upon inspection, reverse sequences were shown to be of insufficient quality and length in some samples, therefore only forward reads were used in downstream analyses with QIIME 2 2020.2 [40]. Forward demultiplexed reads (Casava 1.8 single-end demultiplexed fastq format) were imported to QIIME 2 and summarized using q2-demux plugin followed by denoising with DADA2 q2-dada2 plugin [41]. Forward sequences were trimmed at 5’ end for 10 bp (primer removal) and truncated to 240 bp that produced a final sequence length of 230 bp. DADA2 dereplication produced amplicon sequence variants (ASVs) analogous to 100% operational taxonomic units (OTUs) [42]. ASVs were aligned with mafft [43] (via q2-alignment) and used to construct an unrooted phylogeny tree with fasttree2 [44] (via q2-phylogeny). Taxonomy was assigned to ASVs via q2-feature-classifier [45] classify-sklearn naïve Bayes taxonomy classifier against the SILVA ribosomal RNA sequence database (v. 132) [46]. Mitochondrial and chloroplast sequences were filtered out via q2-taxa prior to calculating alpha and beta diversity metrics via q2-diversity plugin.

Alpha diversity measurements, including Shannon’s diversity index, observed ASVs, and Faith’s phylogenetic diversity, were used for inspecting rarefaction curves to determine suitable sampling depth, and the differences between sampling sites were tested by Kruskal–Wallis H test. Beta diversity analyses were performed on rarefied dataset to 3200 sequences per sample to eliminate bias of different sampling depths [47, 48]. Comparisons of microbial communities were performed through Bray–Curtis, unweighted and weighted UniFrac [49, 50] Principal Coordinate Analyses (PCoA) via q2-diversity plugin. Due to intrinsic compositionality of microbial community datasets obtained by sequencing [51] we used an additional beta diversity analysis on non-rarified data through Robust Aitchison Principal Component Analysis (robust PCA; rPCA) via q2-deicode plugin [52]. Robust PCA is based on centered log-transformation and matrix completion, while retaining feature loadings that may discern between potential microbial niches. The analysis was performed after the exclusion of features with less than ten reads across samples. Log-ratios of rPCA feature loadings were inspected through q2-qurro plugin [53]. The permutational multivariate analysis of variance (PERMANOVA) was used to analyze beta diversity statistical differences via q2-diversity plugin, with the Benjamini–Hochberg false discovery rate (FDR) correction for multiple comparisons. Core features and genera (present in 80% or 85% of samples per sampling site) were determined via q2-feature-table plugin. All plots were visualized with ggplot2 [54] in RStudio (v. 1.3.959) and EMPeror [55].

## Results

A total of 744 531 high-quality reads were obtained for 15 cloacal, 11 oral, and three tank water samples (29 samples in total). The samples had a mean (± SE) 25 673 ± 3 265 sequences per sample that were clustered to 4476 ASVs (Additional file 1). Predominant phyla of cloacal samples consisted of Proteobacteria, Bacteroidetes, Kiritimatiellaeota, Firmicutes and Spirochaetes (> 90% of all cloacal sequences). Oral samples’ predominant phyla were Proteobacteria, Bacteroidetes and Planctomycetes (> 90% of all oral sequences), while tank water exhibited high prevalence of Proteobacteria, Bacteroidetes and Epsilonbacteraeota (> 90% of all tank water sequences). Taxa within phyla varied among individuals, sampling sites, and sampling periods (Additional file 1).

**Fig. 1:**
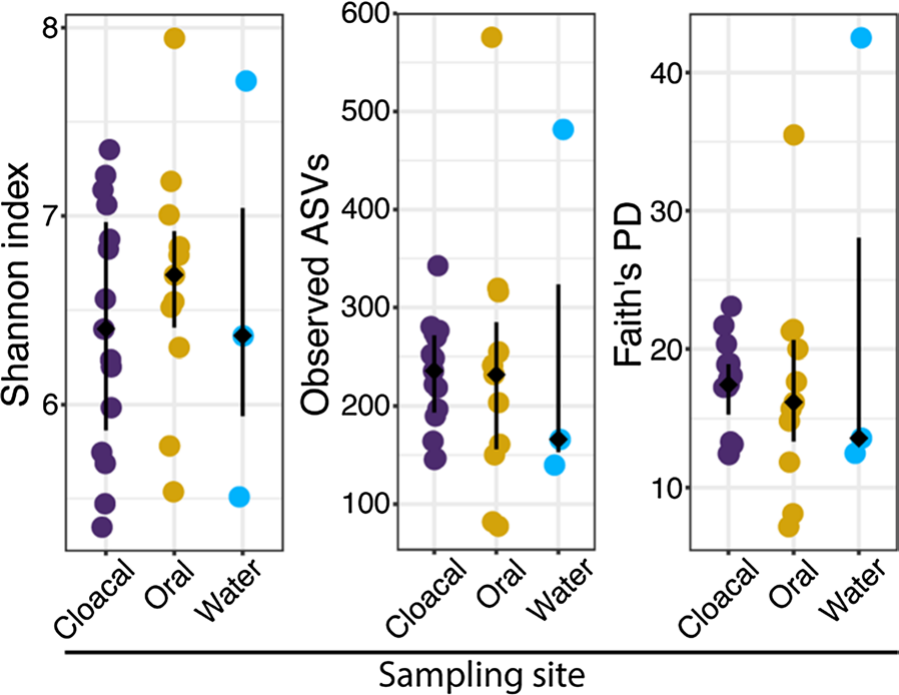
Alpha diversity (Shannon index, observed ASVs, Faith’s Phylogenetic Diversity) of loggerhead cloacal (purple), oral (yellow), and tank water (blue) samples. Filled diamond indicates sample median with lines extending to the upper and lower quartile of sample distribution

Alpha diversity metrics (Shannon’s diversity, observed features, Faith’s PD) were calculated for sampling sites; cloacal, oral, and tank water. No significant difference was observed (*p* > 0.05, Kruskal–Wallis H test) for different sampling sites in either of alpha diversity metrics tested (Additional file 2: Table S2). Tank water did exhibit higher variation than cloacal and oral samples, possibly due to sample size and differences in origin (artificial saltwater in Italy vs. filtered sea water in Croatia that showed greater diversity) (Fig. 1), but it was not significantly different from other sampling sites (Additional file 2: Table S2).

**Fig. 2:**
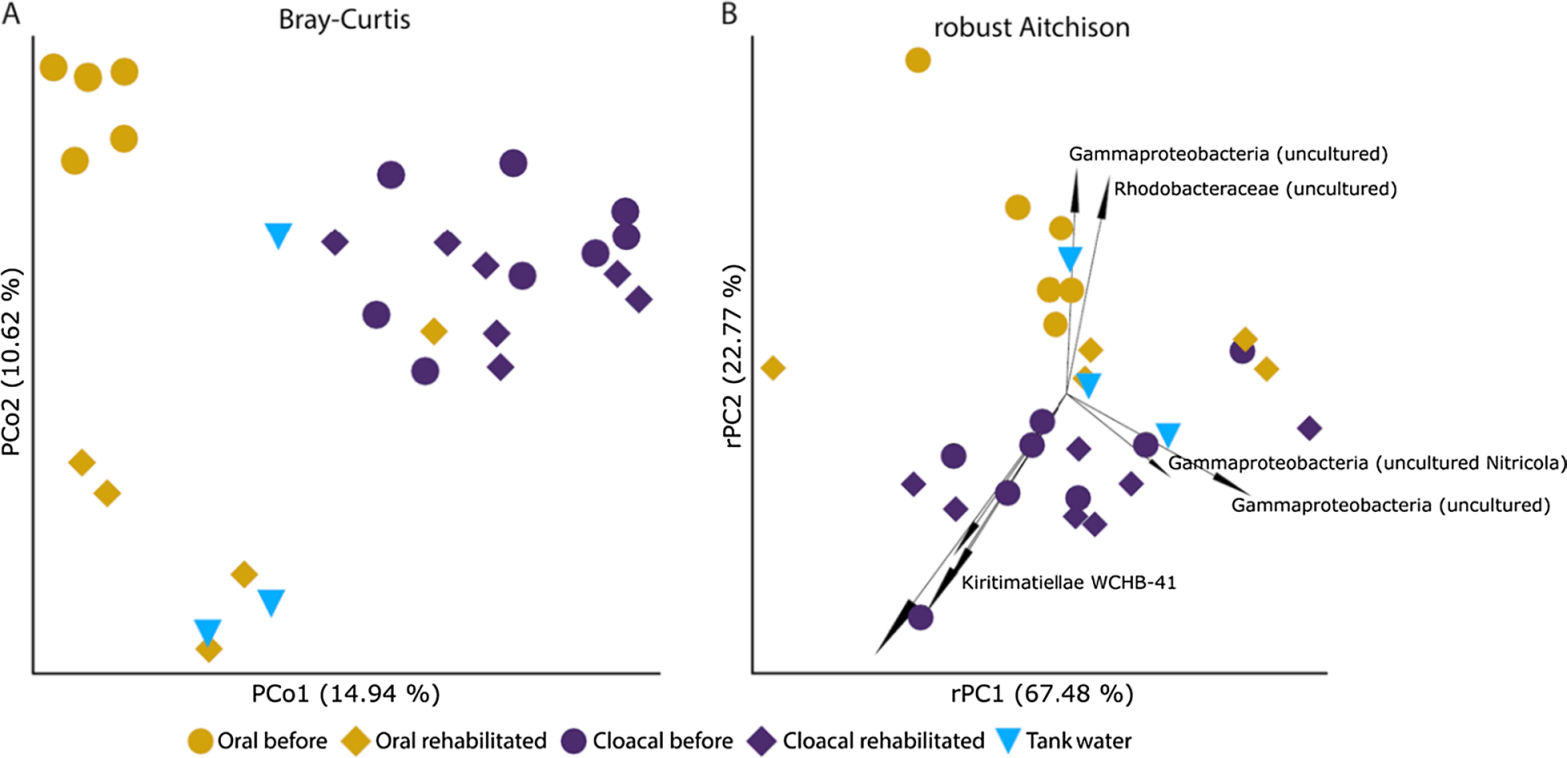
Comparison of microbial diversity in loggerhead cloacal, oral and tank water samples **a** principal coordinate analysis (PCoA) plot of Bray–Curtis distances and **b** principal component analysis (PCA) biplot of robust Aitchison distances with loadings as individual highly ranked ASVs

**Fig. 3:**
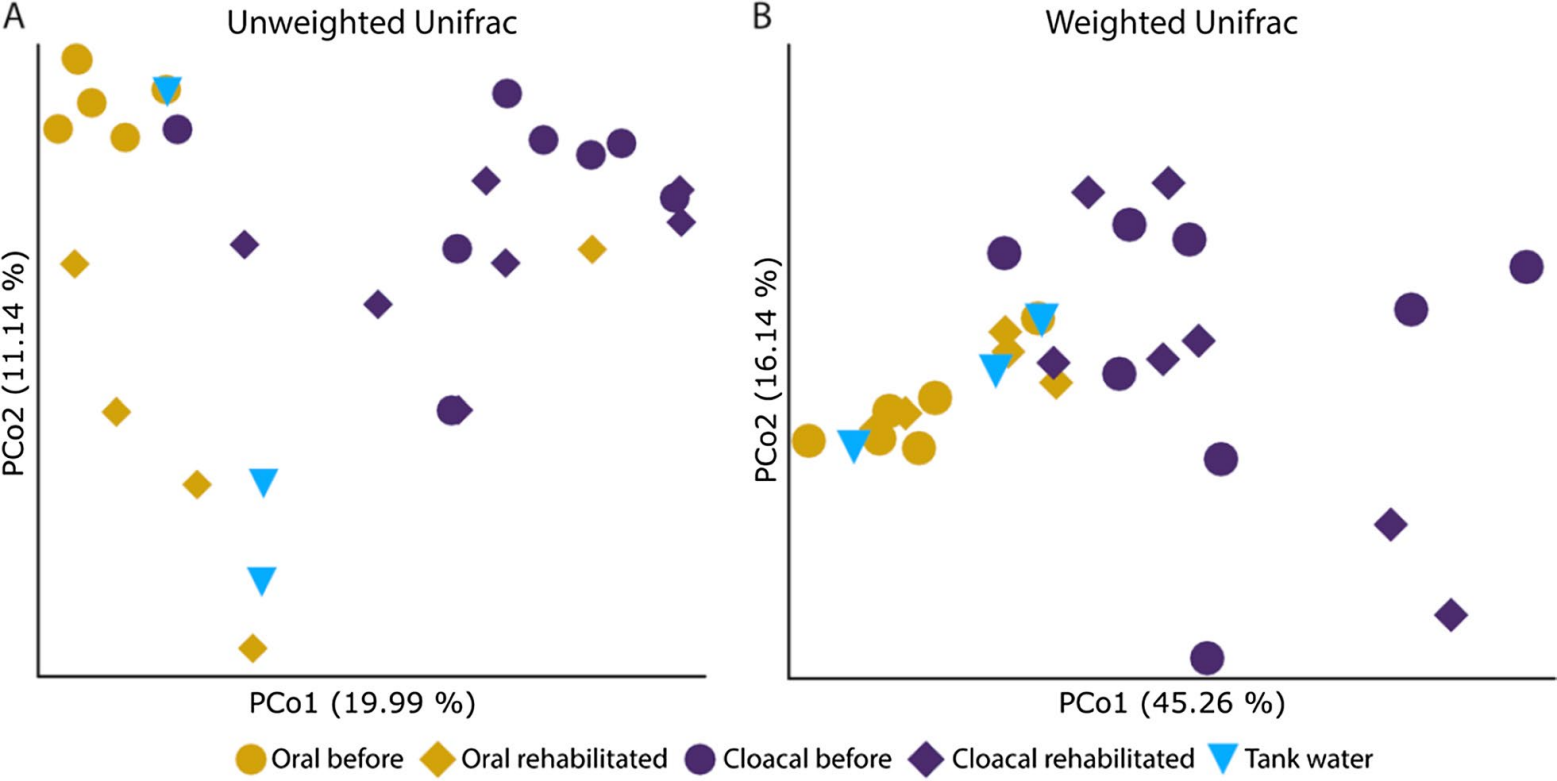
Comparison of microbial diversity in loggerhead cloacal, oral and tank water samples. Principal component analysis (PCoA) plot of **a** Unweighted Unifrac and **b** Weighted Unifrac

Bacterial communities of cloacal samples tended to cluster together, regardless of the sampling period, but oral sample communities showed some separation based on sampling before or during rehabilitation according to PCoA plots (Figs. 2a, 3) and rPCA biplot (Fig. 2b). Tank water samples did not show a visible pattern for Bray–Curtis PCoA or Robust Aitchison PCA (Fig. 2), but for UniFrac PCoA the samples tended to cluster near oral samples (Fig. 3). Feature loadings of Robust Aitchison PCA represent highly ranked individual ASVs, mostly uncultured Gammaproteobacteria, *Rhodobacteraceae*, and members of the Kiritimatiellae WCHB1-41 group (Fig. 2b).

**Fig. 4:**
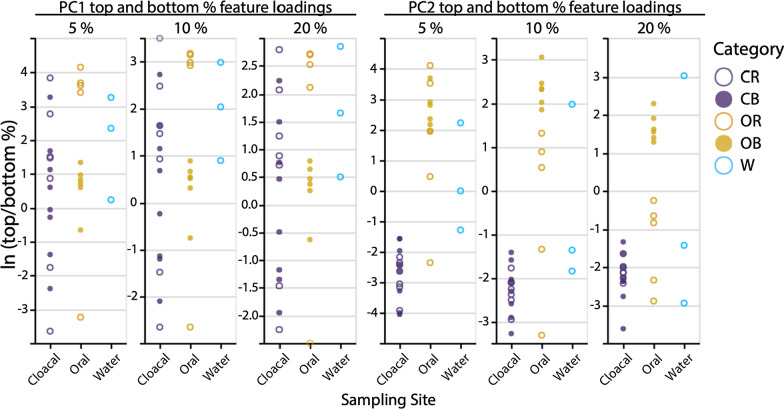
Natural log-ratios (plotted by QURRO) of loggerhead samples’ top and bottom 5%, 10%, and 20% of feature loadings on rPC1 and rPC2 of Robust Aitchison PCA biplot feature loadings by sampling site and period: CB, cloacal before; CR, cloacal rehabilitated; OB, oral before; OR, oral rehabilitated; W, tank water

Based on PERMANOVA (with 999 permutations) bacterial communities differed significantly (*p* < 0.05) between sampling sites and periods (cloacal before, CB; cloacal rehabilitated, CR; oral before, OB; oral rehabilitated, OR; tank water, W) for all used distance metrics tested (Bray–Curtis *p* = 0.001, pseudo-F = 2.37; Robust Aitchison *p* = 0.002, pseudo-F = 3.68; unweighted UniFrac *p* = 0.001, pseudo-F = 2.38; weighted UniFrac *p* = 0.001, psuedo-F = 3.59). Pairwise PERMANOVA testing for sampling site and period groups differed between metrics used with the most conservative result obtained from Robust Aitchison distance that detected a significant difference only between CR versus OB (*p* = 0.005, pseudo-F = 12.27) and CB versus OB (*p* = 0.005, pseudo-F = 10.40). Bray–Curtis distance, unweighted and weighted UniFrac distances pairwise test results showed a significant difference for CB versus OR and OR versus OB in addition to the same sampling site and period conditions that were observed with Robust Aitchison distance pairwise testing. No significant difference was detected between CB and CR samples. Out of all tested metrics, Bray–Curtis and unweighted UniFrac pairwise test results showed a significant difference (*p* < 0.05) among W versus CB, CR, and OB. We detected no significant difference between W and OR samples, which points to the effects of tank water on the oral microbiota of turtles in rehabilitation. Summary of pairwise PERMANOVA tests per distance metric is shown in Additional file 2: Table S3. Visual inspection of natural log ratios of up to 20% top and bottom feature loadings of the Robust Aitchison PCA biplot (Fig. 2b) shows clear segregation of oral before and oral rehabilitated samples (5%, 10%, and 20% top and bottom features on rPC1), and similar log-ratio values of all cloacal samples to oral rehabilitated samples (20% top and bottom features on rPC2) (Fig. 4).

**Fig. 5:**
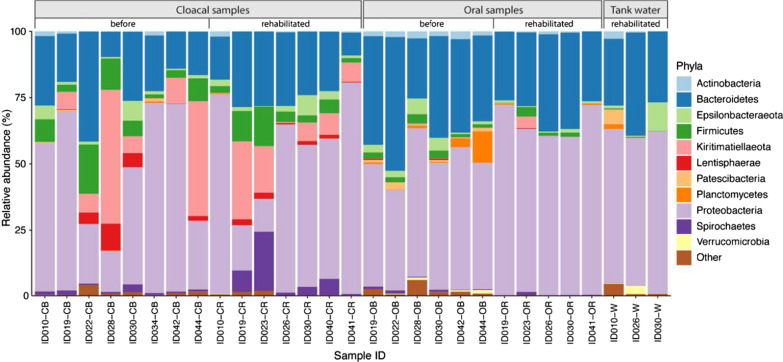
Relative abundances (%) of bacterial phyla present (> 1% on average per sampling site) in loggerhead cloacal, oral and tank water samples. Turtle ID suffix indicates the sampling site and period (before or during/after rehabilitation) as follows: CB, cloacal before; CR, cloacal rehabilitated; OB, oral before; OR, oral rehabilitated; W, tank water

Bacterial communities were distributed across eleven dominant phyla present at > 1% relative abundance in at least one sampling site (Fig. 3). All sampling sites shared dominant phyla Proteobacteria, Bacteroidetes, and to a lesser extent Epsilonbacteraeota (Table 2). Firmicutes were shared between cloacal and oral samples, while tank water and oral samples shared Actinobacteria. Specific to cloacal samples were Kiritimatiellaeota, Spirochaetes and Lentisphaerae phyla, oral samples harbored *Planctomycetes,* and tank water Patescibacteria and Verrucomicrobia (Fig. 5).

**Table 2:** Bacterial phyla of loggerhead sea turtle cloacal and oral samples, and tank water samples from the rescue centers present at > 1% relative abundance on average per sampling site

Bacterial phyla	Cloacal (n = 15)	Oral (n = 11)	Tank water (n = 3)
Actinobacteria	0.52 ± 0.18	**1.37 ± 0.31**	**1.09 ± 0.87**
Bacteroidetes	**21.74 ± 2.16**	**33.88 ± 2.43**	**30.26 ± 4.33**
Epsilonbacteraeota	**2.48 ± 0.63**	**2.02 ± 0.57**	**4.15 ± 3.36**
Firmicutes	**6.74 ± 1.35**	**1.75 ± 0.37**	0.22 ± 0.08
Kiritimatiellaeota	**12.78 ± 4.04**	0.46 ± 0.39	0.03 ± 0.03
Lentisphaerae	**1.99 ± 0.72**	0.14 ± 0.06	0.06 ± 0.03
Patescibacteria	0.17 ± 0.08	0.60 ± 0.24	**1.83 ± 1.83**
Planctomycetes	0.08 ± 0.04	**1.65 ± 1.06**	0.63 ± 0.61
Proteobacteria	**48.60 ± 6.21**	**56.08 ± 3.19**	**58.62 ± 1.56**
Spirochaetes	**3.30 ± 1.47**	0.44 ± 0.14	0.01 ± 0.01
Verrucomicrobia	0.03 ± 0.01	0.30 ± 0.14	**1.14 ± 0.97**

Values represent mean percentage ± SE, with mean values above 1% in bold

Further, bacterial taxa classified to genera (or the next available classification level) present at > 2% relative abundance in at least one sampling site and period conditions indicate taxa specificity to habitat and, on the other hand, the possibility of sharing bacterial taxa of the turtle endomicrobiota with the environment (e.g. Bizionia in oral rehabilitated and tank water bacterial communities) (Table 3). WCHB1-41 taxon (phylum Kiritimatiellaeota) was shown to be almost exclusive for cloacal samples (even though turtle ID010 has not had any sequences of that taxon detected), along with *Treponema* 2, *Aeromonas*, unclassified Aeromonadales, *Desulfovibrio*, unclassified *Rikenellaceae*, and *Bacteroides* genus. Oral samples often shared taxa with cloacal and tank water samples with noticeable differences in relative abundance of *Pseudoalteromonas* and unclassified *Helieaceae* that was not found at > 2% in cloacal samples or tank water. Interestingly, only tank water harbored *Bermanella* as it was not detected in cloacal nor oral samples (Table 3). Based on PERMANOVA results (Additional file 2: Table S3), wild oral samples (before) and oral microbiota during rehabilitation differ significantly, which is also reflected in relative abundances of microbial taxa abundance (Table 3). Wild oral samples harbored more Bacteroidales, *Tenacibaculum*, *Rhodobacteraceae*, Gammaproteobacteria and *Halieaceae*, in comparison to oral samples from turtles in rehabilitation, which showed greater abundance of *Bizionia*, *Pseudoalteromonas*, *Shewanella*, *Pseudomonas*, and *Vibrio,* similar to cloacal and tank water samples (Table 3).

**Table 3:** Bacterial taxa of loggerhead sea turtle cloacal and oral, and tank water samples classified to the genus (or higher taxonomic level) present at > 2% average relative abundance in at least one sampling site and period category (before or wild and during rehabilitation)

Bacterial taxa	Cloacal samples		Oral samples		Tank water
	before (n = 9)	rehabilitated (n = 6)	before (n = 7)	rehabilitated (n = 4)	rehabilitated (n = 3)
Phylum Bacteroidetes					
Bacteroidales; unclassified	1.86 ± 0.60	**2.45 ± 1.07**	**2.21 ± 0.28**	0.19 ± 0.09	0.30 ± 0.24
*Bacteroides*	**2.09 ± 0.61**	1.73 ± 0.76	0.10 ± 0.10	ND	0.13 ± 0.13
*Marinifilum*	1.56 ± 0.55	**4.23 ± 1.87**	1.37 ± 0.54	0.74 ± 0.32	0.54 ± 0.37
*Rikenellaceae*; unclassified	**3.03 ± 1.29**	1.10 ± 0.73	0.14 ± 0.13	ND	0.03 ± 0.03
*Flavobacteriaceae*; unclassified	**2.22 ± 0.88**	1.94 ± 0.33	**13.78 ± 2.93**	**11.54 ± 4.45**	**6.79 ± 1.70**
*Bizionia*	ND	1.29 ± 0.77	0.03 ± 0.03	**11.51 ± 4.27**	**6.25 ± 6.04**
*Flavobacterium*	0.10 ± 0.05	0.84 ± 0.49	**2.23 ± 2.12**	**2.16 ± 1.07**	**5.70 ± 5.26**
*Tenacibaculum*	0.39 ± 0.13	0.50 ± 0.15	**3.44 ± 1.96**	0.81 ± 0.24	**2.77 ± 2.18**
Phylum Kiritimatiellaeota					
WCHB1-41; unclassified	**15.45 ± 6.13**	**8.56 ± 4.26**	0.69 ± 0.61	0.02 ± 0.02	0.03 ± 0.03
Phylum Proteobacteria					
*Rhodobacteraceae*; unclassified	1.69 ± 0.98	1.08 ± 0.24	**8.18 ± 1.63**	**4.45 ± 1.32**	**3.47 ± 1.41**
*Desulfovibrio*	**2.76 ± 0.65**	1.54 ± 0.59	0.37 ± 0.15	0.07 ± 0.07	0.08 ± 0.08
Gammaproteobacteria; unclassified	**11.03 ± 5.02**	**22.69 ± 6.90**	**13.83 ± 2.50**	**3.33 ± 2.01**	**2.96 ± 2.10**
Aeromonadales; unclassified	0.19 ± 0.19	**4.74 ± 4.35**	ND	0.01 ± 0.01	ND
*Aeromonas*	**3.56 ± 3.46**	0.01 ± 0.01	0.44 ± 0.44	ND	0.03 ± 0.03
*Colwellia*	0.21 ± 0.20	0.04 ± 0.03	0.32 ± 0.18	0.81 ± 0.40	**3.29 ± 2.94**
*Pseudoalteromonas*	**2.16 ± 1.55**	**2.94 ± 1.12**	1.70 ± 1.04	**19.52 ± 7.69**	**3.13 ± 2.19**
*Shewanella*	1.54 ± 0.55	**7.15 ± 2.07**	1.76 ± 1.74	**3.86 ± 2.07**	0.77 ± 0.68
*Halieaceae*; unclassified	0.07 ± 0.06	0.03 ± 0.02	**2.16 ± 0.62**	1.68 ± 0.92	0.02 ± 0.02
*Bermanella*	ND	ND	ND	ND	**6.20 ± 6.20**
*Pseudomonas*	0.68 ± 0.33	1.63 ± 0.81	**2.95 ± 2.95**	**5.82 ± 1.99**	**14.10 ± 7.58**
*Vibrio*	**7.24 ± 3.09**	**3.07 ± 1.01**	1.40 ± 0.50	**8.43 ± 4.17**	1.09 ± 0.44
Phylum Spirochaetes					
*Treponema* 2	**3.12 ± 2.29**	**2.22 ± 1.02**	0.12 ± 0.11	ND	ND

Values represent mean percentage ± SE, with mean values above 2% in bold

*ND* not detected

Cloacal samples exhibited two core ASVs (present in more than 85% of samples (12/15)); *Kiritimatiellae* WCHB1-41 and *Treponema* 2. Oral samples did not show any core ASVs at 85% cutoff, but at 80% (8/11 samples) four putative core ASVs were detected, belonging to *Gammaproteobacteria*, *Rhodobacteraceae*, *Pseudoalteromonas*, and *Halieaceae*.

Core taxa collapsed to genus level (present in more than 85% of samples per sampling site) for cloacal samples consisted of uncultured WCHB-41, *Desulfovibrio*, *Bacteroides*, *Shewanella*, *Treponema* 2, *Psychrobacter*, uncultured *Cardiobacteriaceae*, uncultured *Rikenellaceae*, uncultured *Clostridiales* vadin BB60 group, and unassigned *Lachnospiraceae*. Oral samples putative core genera were *Tenacibaculum, Flavobacterium*, and unclassified *Halieaceae*. Genera present both in cloacal and oral samples are *Vibrio*, *Marinifilum*, *Fusibacter* and *Arcobacter* (see Additional file 3).

## Discussion

The results of our research on the microbiota of loggerhead sea turtles show that oral and cloacal microbial communities differ, and that oral microbial assemblages are less stable than cloacal in regard to the turtles’ changing environment (wild *versus* veterinary clinic enclosures). We provide the first insights into oral bacterial communities of incidentally caught wild loggerhead sea turtles and deliver information on how the oral microbiome might respond to short-term rehabilitation in the recovery rescue centers. While most previous studies from the Mediterranean were based on gut microbiome from sick turtles found stranded or dead [31–33] this paper mostly encompasses loggerheads from the wild, incidentally caught during fishing activities. Thus, we consider microbial communities in samples taken prior to admission to the rescue center or clinic as a close representative of the wild microbiome, comparable to recent studies on wild, nesting, adult loggerhead females intestinal microbiome [30, 34]. Only two turtles in this study had to be hospitalized for longer periods due to head injuries (turtle code ID010) or leeches parasitization (turtle code ID023). On the other hand, oral microbiota of sea turtles has not yet been explored by 16S rRNA gene sequencing, while it has been investigated in the freshwater Krefft’s river turtle (*Emydura macquarii krefftii*) and pond slider turtle (*Trachemys s. scripta*) [56, 57].

In our study, alpha diversity metrics did not show significant differences between oral and cloacal body sites or sampling periods (before and during rehabilitation). This could be explained by the size of our target population (relatively small), with juveniles and adults of similar nutritional status, which is insufficient for discovering potential characteristics that could be associated with microbial diversity of samples on this level. In oral microbiomes, ID023 turtle sample is an outlier with much higher microbial diversity, which may be linked with its rehabilitation in the WWF care facility where it was undergoing freshwater treatment for leeches removal prior to admission to the rescue center where it was sampled. Tank water samples from the Aquarium Pula local circulating seawater showed much higher diversity with frequent marine microbial taxa, in comparison to water from the STC in Bari that harbored non-circulating artificial saltwater. Further, aquarium seawater tank exhibited a similar trait to seawater samples in a study by Biagi et al. [33], having a higher diversity of low abundance phyla. Aquarium tank water also had higher abundances of phyla Planctomycetes and Patescibacteria which were observed mostly in oral samples before hospitalization. Therefore, the aquarium recirculating tank water could present a more “natural” marine habitat rather than the tanks with artificial seawater.

Beta diversity metrics consistently showed separation of cloacal and oral microbiomes but with different significance detection between sampling period depending on the metric tested by PERMANOVA. Beta diversity measures used in most sea turtle microbiome studies are still Bray–Curtis dissimilarity, unweighted, and weighted Unifrac even though they do not account for the compositionality of microbiome datasets obtained by sequencing [51]. Due to data compositionality, we decided on presenting already widely accepted beta diversity metrics PCoA along with the robust Aitchison distance PCA,argued to be a better choice for compositional data [52, 58]. Our combined results indicate strong differences between wild cloacal microbiota versus both oral sample periods. Moreover, no significant differences were found among tank water and oral rehabilitated microbiota, which emphasizes the impact of the environment on oral microbiota of loggerhead sea turtles.

Reptile oral microbiomes were considered to be influenced by the prey fecal microbiome but Zancolli et al. [57] observed distinct species-specific patterns in snakes and freshwater turtles that undermine the assumption that reptiles’ oral cavity is a passive reservoir of microbes. As sea turtles are mostly submerged and in close contact with the water medium (sea), we hypothesized that oral microbiome would resemble the environment. As expected, oral samples clustered based on sampling period with samples before rehabilitation clustered closer to the aquarium free-circulating tank water while oral rehabilitated clustered closer to tank water of enclosures with non-filtered artificial seawater (Bray–Curtis and unweighted Unifrac PCoA). No significant differences were observed between oral and tank water samples, but specific bacterial taxa not found in tank water suggest that the oral microbiome consists, at least partially, of endogenous and transitional microbes from the environment. Proteobacteria and Bacteroidetes, abundant in oral microbiota in our study, were also dominantly present in the oral microbiome of Krefft’s river turtle, which was markedly different from the external turtle microbiome and the environment [56]. Comparisons beyond phylum level show that Krefft’s river turtle and pond slider turtle share *Burkholderiaceae* and *Weeksellaceae* families not detected in our study [56, 57] while *Flavobacteriaceae* are shared between Kreffts and loggerheads.

In our study, we detected high abundances of ASVs which could not be classified to genera but only to higher taxonomic ranks: Bacteroidales, *Flavobacteriaceae*, *Rhodobacteraceae*, and Gammaproteobacteria. Highly abundant oral ASVs often overlapped in classification with highly abundant taxa in water tanks, but the actual taxonomic diversity between those groups remains to be determined as overly unclassified reads could implicate a high incidence of yet undiscovered bacteria, or insufficient sequence length required for successful taxonomical identification. Interestingly, the genus *Pseudoalteromonas* was more abundant in oral microbiomes of rehabilitated turtles, while unclassified *Halieaceae* were more abundant in oral microbiomes before rehabilitation than in any other sample type. *Halieaceae* are often found in coastal, neritic environment, deep-sea waters, and in demersal animals (e.g. sponges) [59, 60], hence, they could be easily transported from the marine environment and into the oral cavity. *Pseudoalteromonas* spp. are marine bacteria known for production of antimicrobial substances with many of the species found in association with marine eukaryotes [61] which has been proposed as beneficial to its marine hosts [62]. It is possible that the low abundance taxa in wild oral microbiota are enriched by the veterinary clinic’s enclosure environment conditions; temperature and nutrient availability are relatively stable in comparison to the turtle’s natural habitat. Other taxa that had higher abundances were also notably present in cloacal (*Vibrio* spp., *Shewanella* spp.) or tank water samples (*Pseudomonas* spp., *Bizionia* spp.), which could be transient and non-specific for oral microbiome. At this point, little data are available to compare aquatic turtles’ oral microbiomes beyond the superficial taxonomic levels, and according to our results habitat has a significant effect on the sea turtle oral microbiota. Additional sampling across many different groups of turtles and their habitats would be needed to decouple the effects of the habitat from the intrinsic and possibly representative oral microbes. Even though effects of oral microbial communities on the host have been described in humans and other mammals, it is unknown what roles reptile microbiome may have, especially in marine species [15, 63].

Cloacal microbiome samples did not show any significant clustering of different sample traits in our study design, which is consistent with previous reports [31, 32], but there have been reports on effects of the CCL on cloacal microbiota clustering [33]. As sea turtles often exhibit ontogenetic habitat shift and transit from pelagic to neritic prey, the change in the microbiota regarding to the size and age of the individual could be explained by changed preferences in habitat and food. In juvenile green turtles, there is a significant variation in cloacal microbiomes between pelagic and neritic habitats and transition to herbivorous lifestyle [25]. Additionally, green turtles in rescue centers exhibit a microbiome shift depending on the type of food they receive during rehabilitation, where the fecal microbiome constitutes of bacterial communities prepped for higher protein content as the recovering turtles are fed mostly seafood, but the community shifts to communities known for metabolization of plant polysaccharide upon introduction of plant food near the end of the recovery [24]. The developmental shift from pelagic to neritic habitats of loggerheads in the central Mediterranean Sea is more relaxed, where juveniles have a short epipelagic stage but later choose the habitat opportunistically, according to food availability and oceanographic features [64]. Consequently, shallow north-central Adriatic Sea enables early recruitment to the neritic habitats where rich and diverse benthic pray is available even to small juveniles (< 30 cm) [64]. Presented microbiome of Adriatic Loggerheads seems to confine with satellite tracking and tagging studies that suggest long-term residence of both adults and juveniles in the shallow neritic Adriatic, with seasonal migrations along the Italian coast to the south during winter [65]. Hence, the differences observed in fecal, cloacal and intestinal microbial communities between loggerheads sampled in the central Mediterranean [31, 32], Australia, or USA [30, 34], may be partially explained by highly opportunistic feeding nature and food availability for sampled turtle populations.

The most comprehensive loggerhead microbiota studies from geographically and genetically distinct healthy nesting females [30, 34], usually linked to neritic feeding grounds, reported that microbial communities of the sea turtle gut are dominated by Proteobacteria, followed by Spirochaetes, Bacteroidetes and Actinobacteria. This coincides with our results on wild and early rehabilitation microbial profiles of cloacal samples. On the other hand, microbial communities dominated by higher proportions of Fusobacteria, Firmicutes, and Bacteroidetes with low abundance of Proteobacteria may be considered atypical and describe fecal microbiota of rehabilitated or stranded turtles, connected with the turtle health status [31–33].

The only study on loggerhead microbiome from the Adriatic Sea [33] on fecal microbial communities of stranded or turtles captured in trawling nets showed high abundance of Firmicutes and Fusobacteria, while Bacteroidetes and Proteobacteria were not pronounced. A significant portion of microbial taxa they reported belonged to *Cetobacterium* and *Clostridium* genera, which were not observed in our study. Since these Adriatic loggerheads shared a similar ecological niche and foraging habitats, described non-Proteobacteria dominated microbiome [33] probably arises from their health status, changes in immunity, rehabilitation treatments, recorded period of starvation, and sampling feces rather than the intestine or cloaca. In our study, we detected two putative core cloacal ASVs belonging to WCHB1-41 and *Treponema* 2; while uncultured Clostridiales and *Lachnospiraceae* were detected as putative core taxa and were not overly abundant. Within phylum Bacteroidetes, major components were *Bacteroides,* which have been observed in loggerhead fecal microbiomes [32, 33] and mammalian microbiome [66], *Marinifilum* spp. (commonly found in seawater), unclassified *Rikenellaceae* (specialized for the digestive tract of different animals) [67] and unclassified *Flavobacteriaceae* found in a wide variety of habitats. Interestingly, a major proportion of reads in cloacal samples belonged to the novel Kiritimatiellaeota phylum (formerly in Verrucomicrobia) and were identified as uncultured eubacterium WCHB1-41 [68]. Uncultured WCHB1-41 have been found in equine vaginal and distal gut microbiome, and rumen of cattle [69–71]. Verrucomicrobia have been found in loggerhead cloacal and fecal samples [33, 34] and it is possible that at least a portion of Verrucomicrobia reads would be classified as Kiritimatiellaeota if SILVA v.132 was used to assign the taxonomy, as in this study and study by Arizza et al. [32].

When discussing the representative microbiome of the turtle gut, it is important to discern between the fecal microbiome that is often affected by food composition [24] and is a better descriptor of gut lumen microbiome, *versus* the microbial communities attached to the mucosa and in direct contact with the host, which might or might not be influenced by the shifts in habitat, environment and food type availability [72]. In our study, we used swabs for both oral and cloacal samples rather than feces, as collecting swab samples is less time-consuming in comparison to collecting fecal samples, relatively non-invasive to the turtle and may be performed during fieldwork or within rescue centers. Our results show that cloacal swabs might be sufficient to describe microbial communities as a proxy to feces and intestinal samples, which would allow for wider and less invasive sampling of loggerheads. Sampling wild microbial communities of loggerheads (among other sea turtles and reptiles in general) is necessary to gain basic insights into reptile microbiomes. A recent study in bacterial communities of wild animals via de-novo metagenome assembly showed that wild microbiomes are a resource for novel bacterial species and biological functions [17]. Furthermore, when identifying unknown bacterial genomes of Reptilia microbiota consisted predominantly of novel microbial members and are under sampled in most meta-microbiome studies [6, 9, 17]. Higher abundances of unclassified members of Proteobacteria, Bacteroidetes and other phyla might then prove to be reservoirs of novel bacterial species with interesting features.

Microbial community studies should inform conservation efforts and rehabilitation facilities in ways to improve treatments, housing conditions, and preparation for the release of rehabilitated turtles. In this study, we show that the oral microbiota is potentially less stable and more prone to the acquisition of external microbial taxa in comparison to the relatively stable cloacal microbiota. Implications and effects of long-term rehabilitation of turtles in tanks with non-circulating artificial seawater on the turtles are still unknown and should be investigated further. Due to the sensitivity of oral microbiota to external conditions it should be noted that local circulating seawater should be preferred in rescue centers whenever possible, to preserve and enrich bacterial communities.

## Conclusions

Our work provided the first insights into oral and cloacal microbiota of incidentally caught and mostly healthy loggerhead sea turtles before admission to the rescue center or clinic and after rehabilitation. Other studies focused on hospitalized, dead, and stranded Mediterranean loggerheads [31–33] while our research provided mostly healthy, wild microbiota information as in recent studies on nesting female loggerheads [30, 34]. We showed that cloacal microbiota remains relatively stable during short-term hospitalization, which has been shown in previous studies. Even though loggerhead oral microbial communities do not completely resemble the microbiota of the turtle's environment, they are dynamic and change swiftly as they accommodate taxa from a new environment. Furthermore, cloacal and oral swabs are sufficient for description of microbial communities of loggerheads and allow quick and non-invasive sampling. As reptile microbial communities are still less investigated, wild sea turtle microbiota characterization provides essential information for the expansion of our knowledge on sea turtle biology and guidelines on how to improve on the conservation efforts for these vulnerable, and highly important keystone species in marine ecosystems.

## Supplementary Information

**Additional file 1: Table S1.** Sequencing results (raw counts) and taxonomy assignments per ASV for all samples in this study.

**Additional file 2: Figure S1.** Oral (A) and cloacal (B) sampling of loggerhead sea turtle at the Sea Turtle Clinic of the Department of Veterinary Medicine of University of Bari (Italy). Courtesy of Adriana Trotta. **Table S2.** Alpha diversity measures (Shannon’s diversity, observed ASVs. Faith’s Phylogenetic Diversity) for cloacal, oral and tank water sampling sites with Kruskal–Wallis H test results. Values are represented as mean ± SD, with significance level α < 0.05. **Table S3.** A comparison of differences in microbial communities of different sampling sites and periods by pairwise PERMANOVA for Bray–Curtis, Robust Aitchison, unweighted and weighted UniFrac distance metrics. SampSling sites and periods are marked as follows: CB, cloacal before; CR, cloacal rehabilitated; OB, oral before; OR, oral rehabilitated; W, tank water. P-values shown have been FDR corrected. Significance levels are indicated by an asterisk: *p* ≤ 0.05*, *p* ≤ 0.01** with all significant values bolded.

**Additional file 3: Table S4.** Core taxa of cloacal and oral microbiota at ASV level (85% and 80% cutoff, respectably) and collapsed to the genus level (85% cutoff).

## Data Availability

The amplicon sequencing data obtained in this study are available in the European Nucleotide Archive under accession number PRJEB46638. Additional information about data analysis and extended metadata are available in Mendeley Data repository (10.17632/45skyv5hzy.1).

## Abbreviations

ASV: Amplicon sequence variants
CB: Cloacal before
CCL: Curved carapace length
CR: Cloacal rehabilitated
DNA: Deoxyribonucleic acid
IUCN: International Union for Conservation of Nature
OB: Oral before
OR: Oral rehabilitated
PCoA: Principal coordinate analysis
rPCA: Robust Principal component analysis
rRNA: Ribosomal ribonucleic acid
STC: Sea Turtle Clinic
USA: United States of America
W: Tank water
WWF: World Wildlife Fund
