# Supplementary material for: Characterization of oral and cloacal microbial communities of wild and rehabilitated loggerhead sea turtles (Caretta caretta)
Source: Anim Microbiome. 2021 Sep 3;3:59. doi: 10.1186/s42523-021-00120-5 (PMC8417999; doi:10.1186/s42523-021-00120-5)
Supplement: Supplementary file 2 — Additional file 2: Figure S1. Oral (A) and cloacal (B) sampling of loggerhead sea turtle at the Sea Turtle Clinic of the Department of Veterinary Medicine of University of Bari (Italy). Courtesy of Adriana Trotta. Table S2. Alpha diversity measures (Shannon’s diversity, observed ASVs. Faith’s Phylogenetic Diversity) for cloacal, oral and tank water sampling sites with Kruskal–Wallis H test results. Values are represented as mean ± SD, with significance level α < 0.05. Table S3. A comparison of differences in microbial communities of different sampling sites and periods by pairwise PERMANOVA for Bray–Curtis, Robust Aitchison, unweighted and weighted UniFrac distance metrics. SampSling sites and periods are marked as follows: CB, cloacal before; CR, cloacal rehabilitated; OB, oral before; OR, oral rehabilitated; W, tank water. P-values shown have been FDR corrected. Significance levels are indicated by an asterisk: p ≤ 0.05*, p ≤ 0.01** with all significant values bolded. [file 42523_2021_120_MOESM2_ESM.docx]

**Additional file 2: Figure S1.** Oral (A) and cloacal (B) sampling of loggerhead sea turtle at the Sea Turtle Clinic of the Department of Veterinary Medicine of University of Bari (Italy). Courtesy of Adriana Trotta. **Table S2.** Alpha diversity measures (Shannon’s diversity, observed ASVs. Faith’s Phylogenetic Diversity) for cloacal, oral and tank water sampling sites with Kruskal-Wallis H test results. Values are represented as mean ± SD, with significance level α < 0.05. **Table 3S.** Comparison of differences in microbial communities of different sampling sites and periods by pairwise PERMANOVA for Bray-Curtis, Robust Aitchison, unweighted and weighted UniFrac distance metrics. Sampling sites and periods are marked as follows: CB, cloacal before; CR, cloacal rehabilitated; OB, oral before; OR, oral rehabilitated; W, tank water. P-values shown have been FDR corrected. Significance levels are indicated by an asterisk: p ﻿﻿≤ 0.05*, p ﻿﻿≤ 0.01** with all significant values bolded.

**
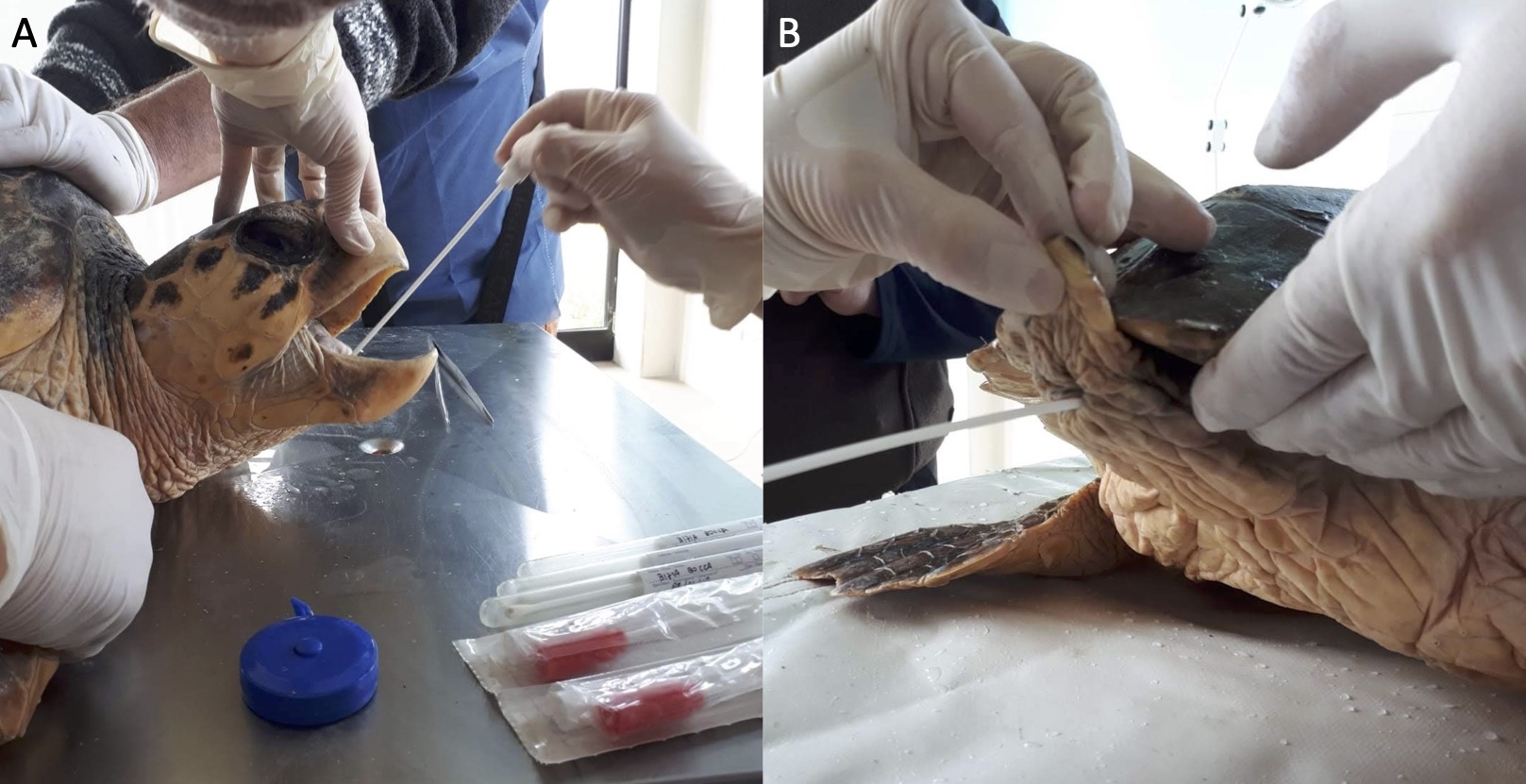
**

**Figure S1.**

**Table S2.**

|  | **Cloacal** | **Oral** | **Tank water** | **p** | **H** |
| --- | --- | --- | --- | --- | --- |
| Shannon | 6.41 ± 0.66 | 6.65 ± 0.66 | 6.53 ± 1.11 | 0.75 | 0.59 |
| Observed ASVs | 230.93 ± 55.44 | 237.64 ± 138.24 | 262.33 ± 189.82 | 0.89 | 0.24 |
| Faith's PD | 17.30 ± 3.27 | 17.24 ± 7.72 | 22.85 ± 17.02 | 0.89 | 0.24 |

**Table S3.**

|  |  | **Bray-Curtis** | | **Robust Aitchison** | | **unweighted UniFrac** | | **weighted UniFrac** | |
| --- | --- | --- | --- | --- | --- | --- | --- | --- | --- |
| **Groups** | **n** | **pseudo-F** | **p-value** | **pseudo-F** | **p-value** | **pseudo-F** | **p-value** | **pseudo-F** | **p-value** |
| CB vs. CR | 15 | 1.120 | 0.284 | 0.200 | 0.931 | 1.094 | 0.278 | 0.492 | 0.804 |
| CB vs. OR | 13 | 2.490 | **0.008**** | 2.280 | 0.186 | 2.426 | **0.016*** | 4.227 | **0.013*** |
| CB vs. OB | 14 | 3.630 | **0.005**** | 10.400 | **0.005**** | 3.890 | **0.003**** | 6.656 | **0.005**** |
| CR vs. OB | 13 | 3.970 | **0.008**** | 12.270 | **0.005**** | 4.020 | **0.003**** | 7.487 | **0.005**** |
| CR vs. OR | 12 | 2.400 | **0.005**** | 2.280 | 0.186 | 1.908 | 0.051 | 4.148 | **0.024*** |
| OB vs. OR | 11 | 2.830 | **0.008**** | 3.450 | 0.153 | 2.167 | **0.003**** | 3.972 | **0.007**** |
| W vs. CR | 10 | 1.870 | **0.008**** | 2.180 | 0.186 | 2.156 | **0.016*** | 2.730 | 0.093 |
| W vs. CB | 11 | 1.840 | **0.011*** | 2.050 | 0.186 | 2.233 | **0.016*** | 2.761 | 0.054 |
| W vs. OR | 8 | 0.940 | 0.424 | 0.060 | 0.971 | 1.040 | 0.405 | 0.691 | 0.804 |
| W vs. OB | 9 | 2.580 | **0.012*** | 3.600 | 0.186 | 2.091 | **0.016*** | 2.324 | 0.054 |
